# Anticholinesterase Toxicity and Oxidative Stress

**DOI:** 10.1100/tsw.2006.38

**Published:** 2006-02-28

**Authors:** Dejan Milatovic, Ramesh C. Gupta, Michael Aschner

**Affiliations:** ^1^ Department of Pediatrics, Medical School, Vanderbilt University, Nashville, TN, USA; ^2^ Toxicology Department, Breathitt Veterinary Center, Murray State University, Hopkinsville, KY, USA

**Keywords:** cholinesterase inhibitors, organophosphates, carbamates, oxidative stress, highenergy phosphate, lipid peroxidation, NMDA

## Abstract

Anticholinesterase compounds, organophosphates (OPs) and carbamates (CMs) are commonly used for a variety of purposes in agriculture and in human and veterinary medicine. They exert their toxicity in mammalian system primarily by virtue of acetylcholinesterase (AChE) inhibition at the synapses and neuromuscular junctions, leading into the signs of hypercholinergic preponderance. However, the mechanism(s) involved in brain/muscle damage appear to be linked with alteration in antioxidant and the scavenging system leading to free radical-mediated injury. OPs and CMs cause excessive formation of F_2_-isoprostanes and F_4_-neuroprostanes, *in vivo* biomarkers of lipid peroxidation and generation of reactive oxygen species (ROS), and of citrulline, a marker of NO/NOS and reactive nitrogen species (RNS) generation. In addition, during the course of these excitatory processes and inhibition of AChE, a high rate of ATP consumption, coupled with the inhibition of oxidative phosphorylation, compromise the cell's ability to maintain its energy levels and excessive amounts of ROS and RNS may be generated. Pretreatment with *N*-methyl D-aspartate (NMDA) receptor antagonist memantine, in combination with atropine sulfate, provides significant protection against inhibition of AChE, increases of ROS/RNS, and depletion of high-energy phosphates induced by DFP/carbofuran. Similar antioxidative effects are observed with a spin trapping agent, phenyl-*N*-*tert*-butylnitrone (PBN) or chain breaking antioxidant vitamin E. This review describes the mechanisms involved in anticholinesterase-induced oxidative/nitrosative injury in target organs of OPs/CMs, and protection by various agents.

## INTRODUCTION

A dramatic increase in the agricultural, industrial, and household use of pesticides over the past several decades has created a growing concern about this class of chemicals. Pesticide residues are now among the most ubiquitous synthetic chemicals in our environment, detectable in the tissues of humans, animals, aquatic life, and wildlife worldwide. Of the wide variety of pesticide agents available, organophosphate

(OP) and carbamate (CM) insecticides are the chemicals most commonly used in the U.S.[1]. In addition, military nerve agents such as soman, sarin, cyclosarin, tabun, and VX are also OP compounds that are chemically related to pesticides, though with greater toxicity. Recent global events have focused attention on the potential threat of international and domestic chemical terrorisms, as well as the possibility of chemical warfare proliferation. Exposure to these agents can lead to both short- and long-term neurological impairments, including: (1) acute hypercholinergic effects that occur minutes or hours following exposure, (2) a delayed intermediate syndrome affecting muscles that can occur within few days following recovery from severe acute affects, (3) a delayed peripheral polyneuropathy associated with some anticholinesterases that usually occurs within weeks following an acute exposure, and (4) subtle, long-term neurological effects, which may last months or even years. Whereas the acute effects are manifest only after a threshold exposure is attained, it has become increasingly apparent that many OPs and CMs can act in a cumulative fashion, with the threshold for more chronic effects reached through repeated exposures at doses that are not associated with acute effects.

Pharmacologically, all these compounds are acetylcholinesterase (AChE) inhibitors, and their acute symptoms are attributed to accumulation of acetylcholine (ACh), thus exhibiting cholinergic toxicity. Phosphorylating or carbamylating the esteratic site of the enzyme[2] diminishes its capacity to catalyze its endogenous substrate ACh. Consequently, the hydrolysis of ACh is prevented, leading to accumulation of ACh in the synaptic cleft and overstimulation followed by desensitization of muscarinic and nicotinic ACh receptors. Depending on the degree of AChE inhibition, cholinergic stimulation may lead to hyperactivity of excitable tissues, causing fasciculations, seizures, convulsions, severe muscle paralysis, hypersecretion from secretory glands, respiratory failure, coma, and death.

A few AChE inhibitors (AChEIs) can also cause another type of toxicity known as organophosphate-induced delayed polyneuropathy (OPIDP). Signs and symptoms include tingling of the hands and feet, followed by sensory loss, progressive muscle weakness and flaccidity of the distal skeletal muscles of the lower and upper extremities, and ataxia[3,4,5]. These occur starting several days (usually 2–3 weeks) after a single exposure, when both cholinergic and intermediate syndrome signs have subsided. Extensive studies carried out in the past 30 years[3,6,7,8,9] have identified the target enzyme, neuropathy target esterase (NTE). Several OPs, depending on their structure, can inhibit NTE, as do some non-OPs, such as CMs and sulfonyl fluorides. For neuropathy to occur, aging of the enzyme must take place and this involves cleavage of the lateral side chain from the phosphorylated NTE and occurs in the axon and not the neuron cell body. These molecular events are then followed by characteristic changes in peripheral nerves, including the degeneration of predominantly long axons, with loss of myelin, and Schwan cell proliferation and macrophage accumulation in nerves. Neuropathy only develops with compounds that are able to inhibit as well as age the NTE.

Chronic organophosphate-induced neuropsychiatric disorder (COPIND)[10] with symptoms of anxiety and depression, memory and attention deficit have also been described in workers exposed to OP compounds. In addition, dystonic reactions, schizophrenia, cog-wheel rigidity, choreoathetosis, and electroencephalographical changes have been reported with high-dose exposures. These extrapyramidal symptoms are thought to be due to the inhibition of the AChE in the human extrapyramidal area. Psychosis, delirium, aggression, hallucination, and depression may also be seen during recovery from the cholinergic syndrome. High levels of anxiety in commercial sprayers of insecticides[11] and other types of delayed neurobehavioral effects are seen among people exposed to low doses of OP compounds for prolonged periods. It is observed that clinical features of psychological syndromes occurring after chronic exposure to OP compounds had great similarity to chronic fatigue syndrome[12].

Increasing evidence suggests that, apart from excessive cholinergic stimulation, activation of glutamatergic neurons is involved in neuronal action of AChEIs. Glutamate release leads to activation of *N*-methyl D-aspartate (NMDA) receptors, massive $Ca^{2+}$ fluxes into the postsynaptic cells, excessive generation of ROS and RNS species, and associated neuronal degeneration. Elevation of cytosolic free $Ca^{2+}$ leads to derangement of many intracellular processes that normally regulate $Ca^{2+}$ availability, including cell energy metabolism[13]. The high intracellular $Ca^{2+}$ concentration is known to damage the mitochondria. In addition, it activates catabolic enzymes, which further affect cell processes[14,15]. The stimulation of other non-NMDA receptors induces recruitment of endogenous $Ca^{2+}$ ions, which further contribute to the induction of the neuronal damage. Glutamate also induces other biochemical mechanisms, which further compromise cellular viability. For example, NMDA activates the synthesis of nitric oxide (NO)[16], a molecule proven to be toxic to neurons and to participate in the formation of seizure activity and neurodegeneration following anticholinesterase intoxication[17].

## Excitotoxicity and Oxidative Stress

Administration of a single sublethal dose of AChEIs induces twitch potentiation, fasciculations, muscular weakness, and acute subjunctional necrosis of muscle fibers. This myopathy can be induced with several OP or CM AChEIs such as paraoxon[18,19,20], diisopropyl phosphorofluoridate (DFP)[21,22,23,24], pyridostigmine[25], aldicarb, and carbofuran (CF)[26], and the OP nerve agents soman, sarin, tabun, and VX[27,28,29]. Despite the diversity in structures of these AChEIs, the induced myopathic changes are the same, suggesting the involvement of a common mechanism. This mechanism invokes excess in ACh in the synapse and prolonged interactions with nAChRs (nicotinic acetylcholine receptors), and not a direct action of these inhibitors on the muscle. The common denominator is muscle hyperactivity, characterized by fasciculations[30].

Seizures, convulsions, and CNS lesions are typical results of systemic application of sublethal doses of AChEIs[31]. The most consistent pathological findings in acute experiments are degeneration and cell death in pyriform cortex, amygdala, hippocampus (where CA1 region is preferentially destroyed), dorsal thalamus, and cerebral cortex. The early morphological changes in AChEI-induced seizures involve dendritic swelling of pyramidal neurons in the CA1 region of the hippocampus[32]. The AChEI-induced neuronal cell death is a consequence of a series of extra- and intracellular events leading to the intracellular accumulation of $Ca^{2+}$ ions and the generation of oxygen-derived free radicals. These, in turn, cause irreversible destruction of cellular components, such as plasmalemma, mitochondria, as well as other intracellular membranes and the cytoskeleton. The increase of seizure-related phosphatidylcholine hydrolysis is likely the cause for the accumulation of free fatty acids and choline in the brain[33].

### *High-Energy Phosphates*

Inhibition of AChE leads to unremitting stimulation of nervous tissue and muscle, which, in turn, causes depletion of high-energy phosphates (HEP), ATP, and phosphocreatine (PCr)[34]. If this stimulation is sufficiently low in intensity or brief in duration, cellular recovery will ensue without lasting consequences. If, however, intense cholinergic stimulation is allowed to persist, a self-reinforcing cycle of cellular damage is set into motion. ATP depletion for several hours to approximately 30–40% of normal levels leads to a fall in the mitochondrial membrane potential that is associated with: (1) reduced energy production (due to decrease in complex I and complex IV activities), (2) impaired cellular calcium sequestration, (3) activation of protease/caspases, (4) activation of phospholipases, (5) activation of nitric oxide synthase (NOS), and (6) excessive generation of ROS. Several of these steps are associated with exacerbation and propagation of the initial depletion of ATP; most notably are the decreases in complex I and IV activities, the impairment of mitochondrial calcium metabolism that regulates ATP production even in the face of a constant supply of substrates, and the generation of nitric oxide, which binds reversibly to cytochrome c oxidase (COx) in competition with oxygen, with subsequent sensitization to hypoxia. COx is the terminal complex in the mitochondrial respiratory chain, which generates ATP by oxidative phosphorylation, involving the reduction of $O_2$ to $H_2O$ by the sequential addition of four electrons and four $H^+$. Electron leakage occurs from the electron transport chain, which produces the superoxide anion radical ($O_2^-$) and $H_2O_2$. Under normal conditions, COx catalyzes more than 90% of the oxygen consumption in the cells. The chance of intermediate products, such as $O_2^-$ and $H_2O_2$ and hydroxyl radical ($OH^-$) escaping is small under conditions where COx remains active. During the hyperactivity of brain or muscle, the activity of COx is reduced[35,36], leading to an increased electron flow within the electron transport chain, thereby increasing ROS generation, oxidative damage to mitochondrial membranes, and increasing vulnerability to excitotoxic impairment[36,37,38,39,40,41]. We have previously established that several of these key events occur within 1 h of anticholinesterase treatment[42]. DFP-induced seizures markedly lowered the cellular ATP and PCr levels in discrete brain regions[43,44] and significantly reduced COx activity[36].

Data on HEP and their metabolites in the brain regions of control rats and those intoxicated with DFP (1.25 mg/kg, s.c.) or CF (1.25 mg/kg, s.c.) are shown for amygdala and hippocampus in Fig. 1. Control values of energy metabolites were not significantly different in the amygdala and hippocampus. One hour after DFP or CF, the levels of ATP, TAN, PCr, and TCC were significantly reduced in both brain regions. Three days after anticholinesterase treatment, significant recovery of ATP and PCr was observed in cerebrum.

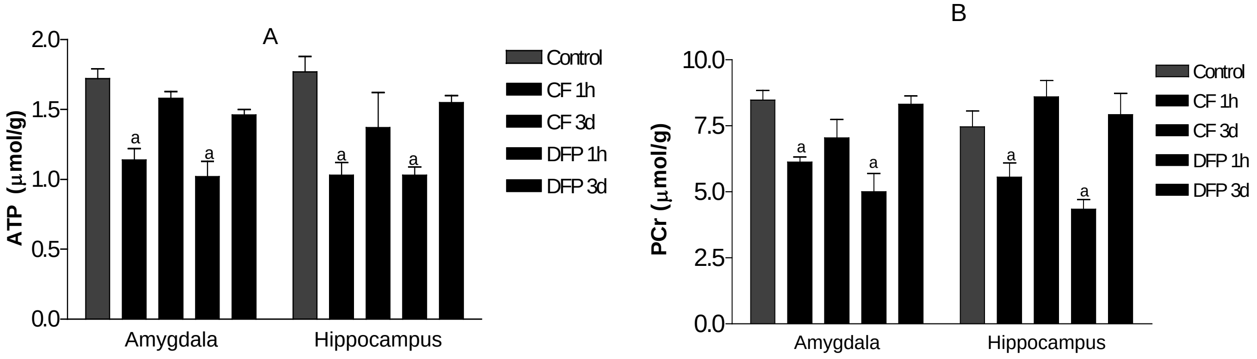

**FIGURE 1.** Levels of high-energy phosphates, (A) ATP and (B) PCr in amygdala and hippocampus of rats intoxicated with an acute dose of CF (1.25 mg/kg, s.c.) or DFP (1.25 mg/kg, s.c.). Rats were sacrificed 1 h or 3 days (3d) after CF or DFP injection. Values of ATP and PCr are presented as mean ± SEM (n = 4–6). [a]Significant difference between values from control rats and DFP- or CF-treated rats ($p < 0.05$).

During the course of these excitatory processes, a high rate of ATP consumption, coupled with the inhibition of oxidative phosphorylation, compromises the cell's ability to maintain its energy levels, and excessive amounts of ROS and RNS may be generated. Thus, the combination of impaired synthesis of ATP with its greater utilization during brain hyperactivity appears to result in a significant depletion of ATP.

## Role of NO/NOS

A major stimulus for NO production is elevation of intracellular $Ca^{2+}$, which binds to calmodulin, resulting in the activation of NO synthase. NO is a labile RNS endowed with messenger functions[45]. Increases in intraneuronal $Ca^{2+}$ stimulate neuronal NO synthase (nNOS), which oxidizes L-arginine with stoichiometric production of L-citrulline and NO[46]. Its role in convulsive effect has been studied in different experimental models, though the reported results are far from unequivocal. For example, the proconvulsive activity of NO in the seizures induced by the excitatory NMDA, as well as by the AChEIs, has been demonstrated[17,47,48,49,50,51,52,53,54]. Conversely, the results of other studies indicate that NO may play the role of an endogenous anticonvulsant substance[55,56,57]. Part of this controversy, regarding NO's neurotoxic vs. neuroprotective actions[58] may be due to the redox state of NO or the nitrosonium ion ($NO^-$). NO can cause neurotoxicity by reacting with the superoxide $O_2^-$, leading to the formation of $OONO^-$[58]. Whether the seizure-induced increase in NO has proconvulsive or anticonvulsive actions may depend also on the amount of NO generated by NOS.

Many reports provide evidence that NO impairs mitochondrial/cellular respiration and other functions by inhibiting the activities of several key enzymes, particularly COx, and thereby causing ATP depletion[34,36,41,59]. Inhibition of COx appears to be the primary mechanism for ATP depletion. This is further supported by the findings that AChE-inhibiting OP insecticides cause inhibition of oxidative phosphorylation in the rat brain[60].

Data on citrulline levels (an indicator of NO/NOS activity) in muscle of control rats and those intoxicated with an acute dose of CF (1.5 mg/kg, s.c.) are shown in Fig. 2. Analyses of controls revealed slightly higher citrulline levels in the soleus (469.74 ± 31.81 nmol/g) than in the extensor digitorium longus EDL (417.84 ± 18.54 nmol/g). Within 15 min after exposure to CF (1.5 mg/kg, s.c.), citrulline levels were significantly elevated in both muscles (soleus, 155%; EDL, 176%). Maximum increases in citrulline levels were noted after 1 h (267 and 304%, respectively). Levels remained significantly elevated until 6 h and returned to control levels when measured after 24 h.

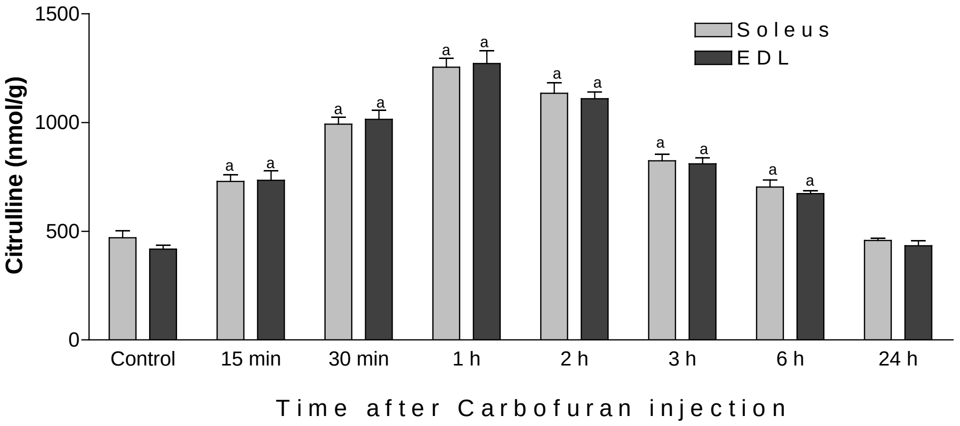

**FIGURE 2**. Levels of citrulline in soleus and EDL muscles of rats intoxicated with an acute dose of CF (1.5 mg/kg, s.c.). Values of citrulline are presented as mean ± SEM (n = 4–6). [a]Significant difference between control and CF-treated rats ($p < 0.05$).

Within 5 min of CF injections, citrulline levels were elevated more than 4-fold in the cortex and more than 2-fold in the amygdala and hippocampus (Fig. 3). The highest levels of citrulline were achieved 30 min after CF injection. The maximal increases in citrulline in cerebrum (approximately 6-fold in cerebral cortex) compared to skeletal muscle (approximately 2.5-fold), the earlier attainment of peak citrulline levels (30 min in cerebrum vs. 1 h in skeletal muscle), and the persistence of elevated cortical citrulline 24 h after injection are likely manifestations of higher sensitivity of cerebrum compared to skeletal muscle to effects of anticholinesterases on RNS.

## *Lipid Peroxidation and* In Vivo *Markers of AChEI–Induced Oxidative Damage*

Due to high concentration of substrate polyunsaturated fatty acids in cells, lipid peroxidation is a major outcome of free radical-mediated injury[61,62]. A critically important aspect of lipid peroxidation is that it will proceed until oxidizable substrate is consumed or termination occurs, making this fundamentally different from many other forms of free radical injury in that the self-sustaining nature of the process may entail extensive tissue damage[63]. Two broad outcomes of lipid peroxidation are structural damage to cellular membranes and generation of oxidized products, some of which are chemically reactive and may covalently modify cellular macromolecules. It is these reactive products that are thought to be the major

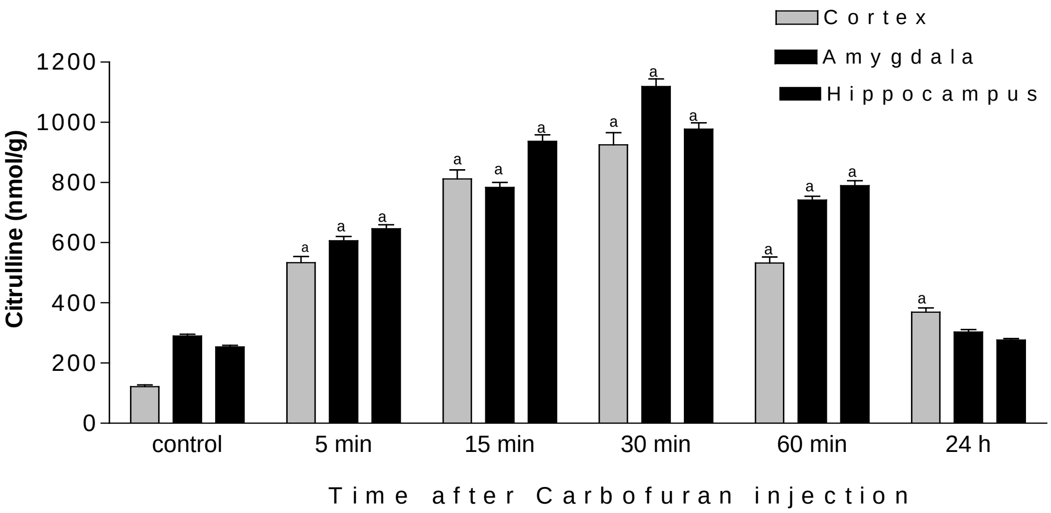

**FIGURE 3.** Citrulline levels in brain regions of rats intoxicated with an acute dose of CF (1.25 mg/kg, s.c.). Values of citrulline are presented as mean ± SEM (n = 4–6). [a]Significant difference between control and CF-treated rats ($p < 0.05$).

effectors of tissue damage from lipid peroxidation[64]. Biochemical studies have demonstrated increased concentrations of reactive products from lipid peroxidation in neurodegenerative disease including Alzheimer's disease[65]; however, a few studies have investigated the presence or significance of these markers of oxidative stress in muscle and nervous tissue in anticholinesterase-induced toxicity.

The use of reactive products of lipid peroxidation as *in vivo* biomarkers is limited because of their chemical instability and rapid and extensive metabolism[66,67]. For these reasons, other more stable lipid products of oxidative damage have generated intense interest in recent years as *in vivo* markers of oxidative damage. These compounds include the $F_2$-isoprostanes ($F_2$-IsoPs), isofurans (IsoFs), and $F_4$-neuroprostanes ($F_4$-NeuroPs)[68,69]. $F_2$-IsoPs are formed by peroxidation of arachidonic acid (AA). Three major structural isomers of IsoPs are formed from a common intermediate: F-ring IsoPs formed by reduction and D/E-ring IsoPs formed by isomerization[70]. The ratio of F-ring to D/E-ring compounds reflects the reducing environment in which $F_2$-IsoPs form, with greater reducing equivalents favoring a higher ratio of F- to D/E-ring compounds[70]. More recently, it has been shown that in the presence of increased oxygen tension in the microenvironment in which lipid peroxidation occurs, an additional oxygen insertion step may take place[69]. This step diverts the IsoP pathway to form tetrahydrofuran ring-containing compounds termed IsoFs, which are functional markers of lipid peroxidation under conditions of increased oxygen tension.

Similar studies of lipid peroxidation products have been performed for other substrate lipids. Of particular interest are oxidation products of docosahexaenoic acid (DHA), which have been termed $F_4$-NeuroPs. In contrast to AA, which is evenly distributed in all cell types in all tissues, DHA is highly concentrated in neuronal membranes[71]. Thus, determination of $F_4$-NeuroPs permits the specific quantification of oxidative damage to neuronal membranes *in vivo*. In fact, to our knowledge, $F_4$-NeuroPs are the only quantitative *in vivo* marker of oxidative damage that is selective for neurons.

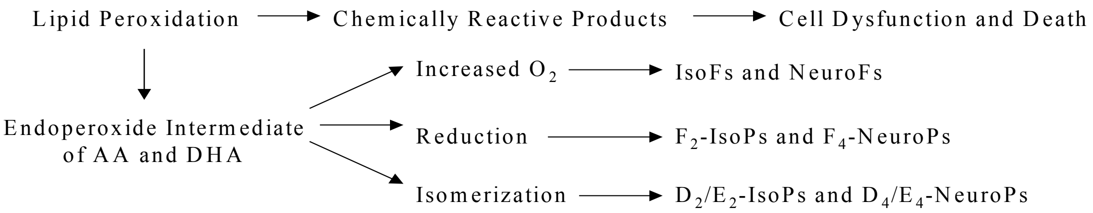

A significant increase in $F_2$-IsoPs was seen in EDL within 60 min following CF injection (Fig 4). $F_2$-IsoPs levels in EDL returned to control levels within 6 h following CF injection. Also, rats receiving DFP or CF showed typical signs of AChE toxicity, including tremors, wet dog shakes, mild to moderate seizures and convulsions, with rearing and falling over, progressing to severe seizures within 15–30 min. Significant increases in $F_2$-IsoPs and $F_4$-NeuroPs were seen in brain within 30 min following DFP or CF injection (Fig. 5), indicating increased lipid peroxidation. The induced changes were rapid and returned to control levels within 2–3 h.

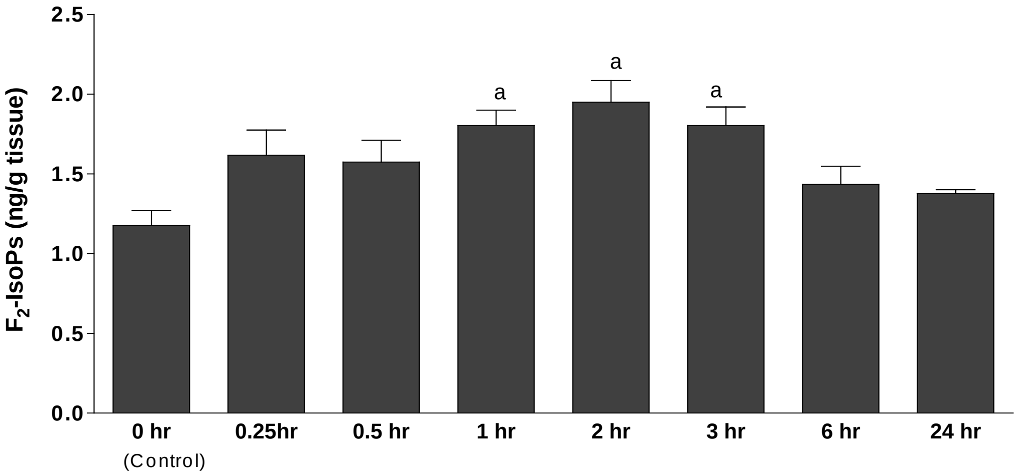

**FIGURE 4.** Effect of CF (1.5 mg/kg, s.c.) on $F_2$-IsoPs levels in EDL muscles. Values are mean ± SEM (n = 4–6). [a]Significant difference between control and CF-treated rats ($p < 0.05$).

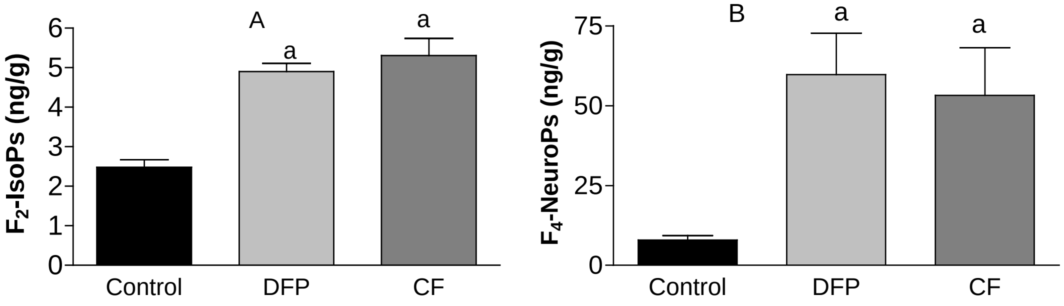

**FIGURE 5**. Effect of DFP (1.5 mg/kg, s.c.) or CF (1.5 mg/kg, s.c.) on (A) $F_2$-IsoPs and (B) $F_4$-NeuroPs levels in rat brain. Values are mean ± SEM (n = 4–6). [a]Significant difference between control and DFP- or CF-treated rats ($p < 0.05$).

## Prevention of AChEI-Induced Oxidative Stress

### *Spin-Trapping Agents*

Electron spin resonance (ESR) spectroscopy using spin traps allows direct identification and characterization of ROS. A synthetic spin-trapping agent such as phenyl-*N*-*tert*-butylnitrone (PBN) is capable of scavenging many types of free radicals. This compound is widely used to trap ROS in a variety of physical, chemical, and biological studies using electron paramagnetic resonance spectrometry. PBN is known to be concentrated in the mitochondria, where it reacts with ROS and forms stable adducts, and thereby maintains normal levels of energy metabolites. Numerous *in vitro* and *in vivo* experiments have shown the beneficial effects of PBN on the prevention of neuronal degeneration. Protective effects are described in experimental models of brain ischemia/reperfusion[72,73,74,75,76,77,78], excitotoxicity[79, 80,81], 3,4-methylenedioxy-methamphetamine (MDMA) intoxication[82], and in different models of seizures[83,84]. These findings led to speculation that PBN might be a useful tool in preventing neurodegeneration in Parkinson's[85,86] and Alzheimer's disease[87]. Other pharmacological properties of spin-trapping agents have been described that could influence the outcome of oxidant injury. These have been described for PBN as reversible $Ca^{2+}$ channel blockade in vascular muscle causing vasodilatation[88], direct effects on seizures[89], direct effect on striatal function, including inhibition of excitation-contraction coupling[90], inhibition of AChE activity[91,92], induction of hypothermia[93], and inhibition of nitric oxide synthase induction[94,95].

Pretreatment with PBN (300 mg/kg, i.p.) prevented muscle hyperactivity as well as necrosis and attenuated the DFP-induced AChE inhibition otherwise seen in DFP-only treated rats. PBN had no effect when given after fasciculations were established. While the role of PBN as an antioxidant is well established, its prophylactic effect against excitotoxity induced by an AChEI is due to its protection of AChE from critical inhibition, an unexpected action[91,92].

The spin-trapping agent PBN prevented DFP- or CF-induced convulsions and seizures. This could primarily be due to a protective interaction of PBN with AChE, sufficient to protect a critical fraction of AChE against phosphorylation by DFP or carbamylation by CF[89,91,92].

PBN pretreatment 1 h before DFP prevented seizures, protected mitochondria, and maintained cellular level of high-energy metabolites (Fig. 6). Protection was also seen with vitamin E pretreatment, a naturally occurring antioxidant. Vitamin E pretreatment did not prevent DFP-induced seizures. PBN or vitamin E treatment alone did not alter the levels of high-energy phosphates or their major metabolites in any of the brain regions (data not shown). The findings in Fig. 6 demonstrate that AChEI-induced energy depletion of metabolites are, in part, also prevented by antioxidants (PBN or vit E), supporting the suggestion that increased generation of ROS/RNS contribute to depletion of energy phosphates[59,96].

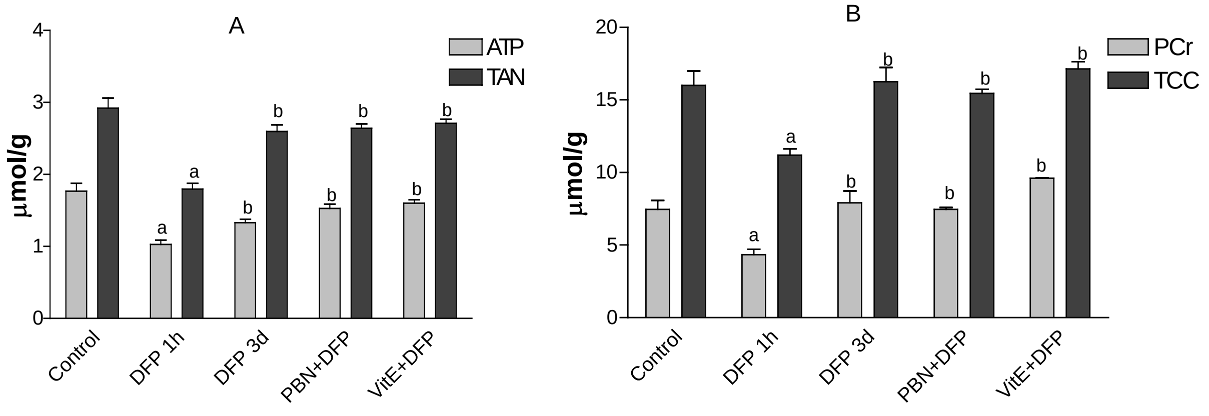

**FIGURE 6.** Effects of vitamin E (VitE) and N-tert-butyl-α-phenylnitrone (PBN) on DFP-induced changes on high-energy phosphates, (A) ATP and TAN and (B) PCr and TCC in hippocampus. Values are presented as mean ± SEM (n = 4–6). TAN = total adenine nucleotides (ATP+ADP+AMP); TCC = total creatine compounds. [a]Significant difference between control and treated groups ($p < 0.05$). [b]Significant difference between DFP 1 h and other treated groups ($p < 0.05$).

The data presented in Fig. 7 show that AChEI-induced increases in NO (citrulline) were significantly prevented by PBN as well as by vitamin E. There is evidence that suggests that PBN inhibits the induction of inducible NOS (iNOS) by reducing the expression of iNOS protein (decrease in mRNA expression), and thus prevents the overproduction of NO[95,97].

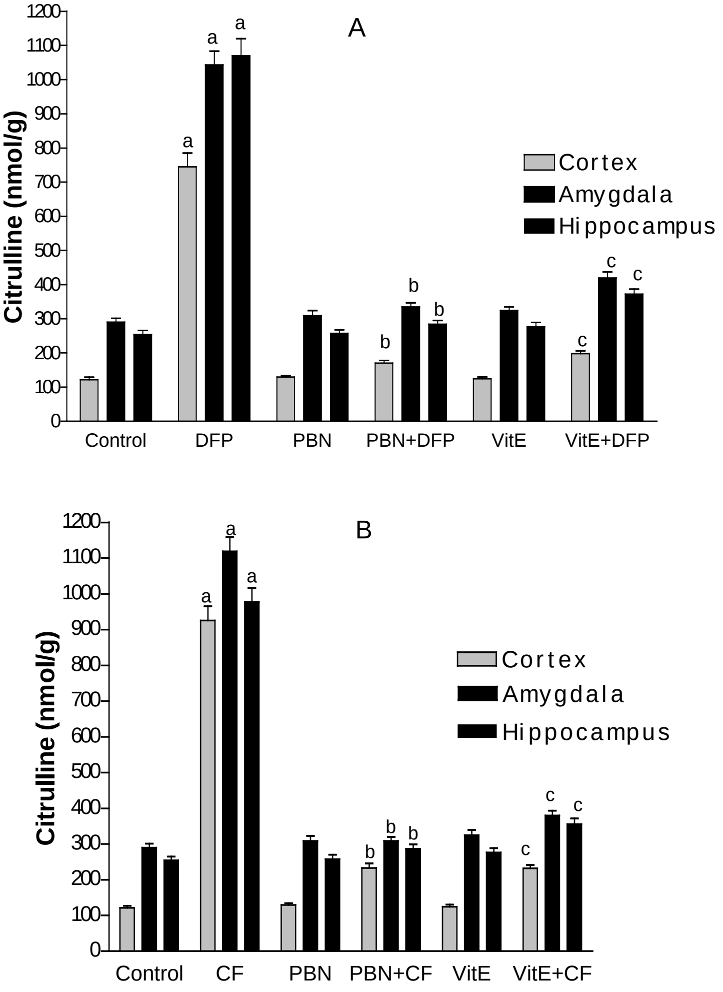

**FIGURE 7.** Protection for (A) DFP- or (B) CF-induced changes in citrulline levels in rat brain regions by antioxidant treatment with PBN or Vit E. Values of citrulline are presented as mean ± SEM (n = 4–6). [a]Significant difference between values from control rats and DFP- or CF-treated rats ($p < 0.05$), [b]Significant difference between values from DFP- or CF-treated rats and PBN+DFP– or PBN+CF–treated rats ($p < 0.05$), [c]Significant difference between values from DFP- or CF-treated rats and VitE+DFP– or VitE+CF–treated rats ($p < 0.05$).

## Antioxidants

Reduction in ATP and PCr, increase in citrulline levels, as well as the loss of COx activity can be attenuated by antioxidants. Vitamin E pretreatment suppressed the depletion of HEP and their metabolites and increase in citrulline levels without preventing seizures (Figs. 6 and 7). Previous studies have shown that mitochondria contain the highest concentration of vitamin E[98] and accelerate ATP resynthesis in tissues subjected to ischemia/reperfusion[99]. Vitamin E also prevented metasystox (OP insecticide)–induced changes in lipase activity and lipid peroxidation in the brain and spinal cord of rats[100]. Vitamin E mainly acts as a chain-breaking antioxidant and radical scavenger, protecting cell membranes against oxidative damage[101]. In addition, vitamin E regulates ROS production[102], maintains oxidative phosphorylation in mitochondria, and accelerates restitution of high-energy metabolites[99,103]. The protection provided by vitamin E against DFP- or CF-induced changes in energy metabolites was of varying degrees in different brain regions and could partly be due to pharmacokinetic variables involved in attaining different levels of vitamin E in different brain regions. Spin-trapping agents and antioxidants like vitamin E, either by preventing seizure activity and/or by scavenging ROS, provide partial protection against depletion of energy metabolites, maintain adequate cellular energy status, and thus diminish neuronal and muscle injury[34,36,104].

## Memantine

*In vivo* experiments conducted in rats established that preadministration of memantine, an uncompetitive NMDA-receptor antagonist, significantly protected AChE activity from inhibition caused by AChEIs, including OP and CM insecticides[105,106,107] and the OP nerve agents[108,109]. Studies published by us and others revealed that memantine exerts various pharmacological effects by multiple pharmacological mechanisms. In brief, these mechanisms include: (1) blockage of nicotinic acetylcholine receptor-ion channel complex[110], (2) reduced reflex excitability of both flexors and extensors[111], (3) prevention of neural hyperexcitability[109], (4) reduced high-frequency repetitive activation of peripheral nerves[112], (5) central muscle relaxation[113], and (6) prevention of AChEI-mediated energy loss from muscle cells[114].

Pretreatment of rats with memantine (MEM) and atropine sulfate (ATS), 60 and 15 min, respectively, before DFP administration, provided complete protection against DFP-induced behavioral changes, since no muscle fasciculations were seen at any time. MEM in combination with ATS, in the same dose (18 mg/kg, s.c.), did not produce any untoward effects in DFP-untreated rats. One hour after DFP injection, when rats exhibited signs of maximal severity, AChE activity in skeletal muscles was reduced by 90–96%. No significant change occurred in the enzyme activity in muscles of rats (DFP-untreated) receiving MEM and ATS. However, pretreatment of animals with MEM in combination with ATS provided significant protection of AChE (soleus 31%, EDL 32%, and diaphragm 44%) against AChE-induced inactivation and depletion of high-energy phosphates (Fig. 8) in skeletal muscles. Prophylactic administration of MEM and ATS also blocked the AChEI-induced increase in levels of citrulline (Fig. 9) and $F_2$-IsoPs (Fig. 10), markers of NO synthesis and lipid peroxidation[115], respectively. These data support findings that MEM exerts various pharmacological effects by multiple pharmacological mechanisms.

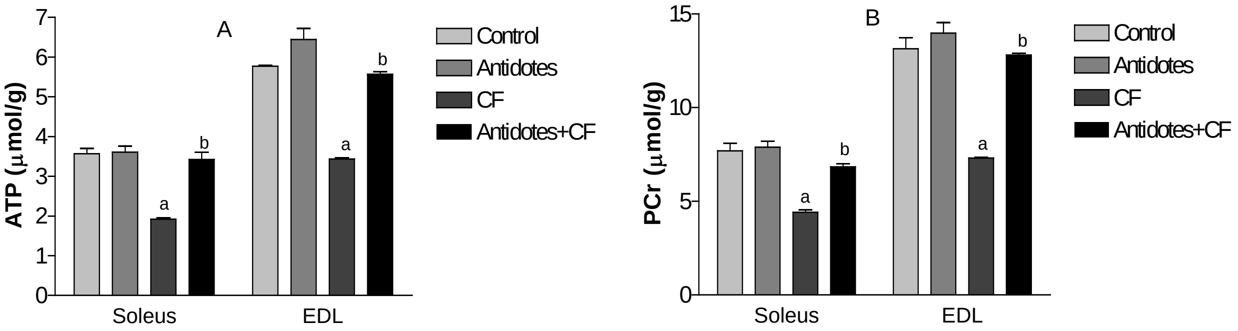

**FIGURE 8.** Protection by antidotes (MEM, 18 mg/kg and ATS, 16 mg/kg) of CF (1.5 mg/kg)–induced changes in high-energy phosphates (A) ATP and (B) PCr in soleus and EDL muscles of rats. Animals were sacrificed 60 min after last injection. Each value presented as mean ± SEM (n = 4–6). [a]Significant difference between values from controls and treated rats ($p < 0.05$). [b]Significant difference between values from CF and Antidotes+CF–treated rats ($p < 0.05$).

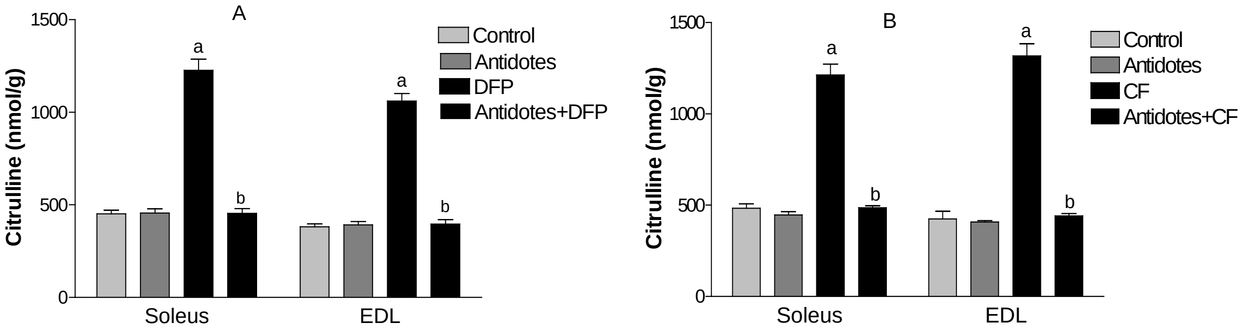

**FIGURE 9.** Effects of antidotes (MEM, 18 mg/kg, s.c. and ATS, 16 mg/kg, s.c.), given prophylactically 60 and 15 min, respectively, before (A) DFP or (B) CF administration on citrulline levels in skeletal muscles soleus and EDL of rats. Rats were sacrificed 60 min after DFP or CF injection. Values of citrulline are presented as mean ± SEM (n = 4). [a]Significant difference between values from control rats and DFP- or CF-treated rats ($p < 0.05$). [b]Significant difference between values from DFP- or CF-treated rats and Antidotes+DFP– or Antidotes+CF–treated rats ($p < 0.05$).

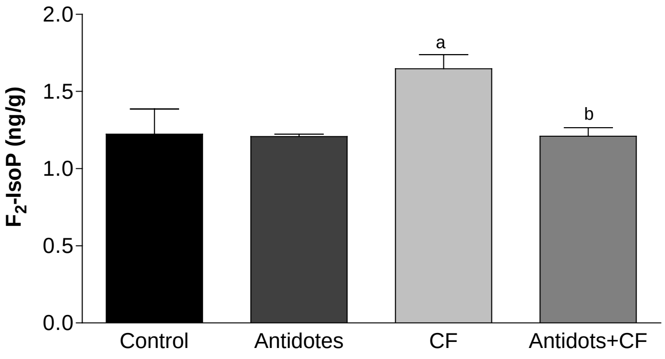

**FIGURE 10.** Effects of antidotes (MEM, 18 mg/kg and ATS, 16 mg/kg) given prophylactically, 45 and 15 min, respectively, before CF (1.5 mg/kg, s.c.) administration on $F_2$-IsoPs levels in EDL muscles. Animals were sacrificed 60 min after last injection. Values are mean ± SEM (n = 4–6). [a]Significant difference between values from control and CF-treated rats ($p < 0.05$). [b]Significant difference between values from CF- and Antidotes+CF–treated rats ($p < 0.05$).

## CONCLUSIONS

The AChEIs continue to be important economic poisons, environmental contaminants, and therapeutic agents, and they are of concern for chemical terrorism, due in large part to their capacity to affect a variety of CNS functions. *In vitro* and *in vivo* data strongly implicate free radical-mediated injury to intoxicated and diseased regions of the brain as a shared mechanism among several neurodegenerative diseases, highlighting the susceptibility of nervous tissue, with its high metabolic activity and limited regenerative potential, to this type of insult. While free radical damage may be a shared mechanistic aspect of toxicity among a variety of neurodegenerative diseases, the sources of free radical damage likely are specific to different types of neurodegeneration and toxicant exposure.

The CM and OP anticholinesterases induce neuro- and myotoxic effects that destroy neurons and muscle fibers by excitotoxic action. Additional noncholinergic mechanisms that contribute to these effects include excessive generation of free radicals and alteration in antioxidant and the scavenging system, causing lipid peroxidation. Understanding the relationships of excitotoxicity, cholinergic, and noncholinergic (oxidants/antioxidants) determinants may elucidate biochemical mechanisms that are crucial to a variety of toxicities of cholinesterase inhibitors, and relate important aspects of their toxicity to an established conceptual framework of other neurodegenerative disease.

## ACKNOWLEDGMENTS

This work was supported in part by Forest Laboratories, Inc. research contract NAM 38. The authors thank Snjezana Zaja-Milatovic for her assistance in the preparation of the manuscript.

The authors also acknowledge the kind permission of Springer Science and Business Media and Elsevier for the following: Figs. 3 and 7, adapted from Gupta, R.C., Milatovic, D., and Dettbarn, W.-D. (2001) Nitric oxide modulates high-energy phosphates in brain regions of rats intoxicated with diisopropylphosphorofluoridate or carbofuran: prevention by N-tert-butyl-alpha-phenylnitrone or vitamin E. *Arch. Toxicol.* **75**, 346–356 (with permission from Springer Science and Business Media); Figs. 4 and 10, adapted from Milatovic, D., Gupta, R.C., Dekundy, A., Montine, T.J., and Dettbarn, W.-D. (2005) Carbofuran-induced oxidative stress in slow and fast skeletal muscles: prevention by memantine and atropine. *Toxicology* **208**, 13–24 (with permission from Elsevier).
